# Performing optical logic operations by a diffractive neural network

**DOI:** 10.1038/s41377-020-0303-2

**Published:** 2020-04-13

**Authors:** Chao Qian, Xiao Lin, Xiaobin Lin, Jian Xu, Yang Sun, Erping Li, Baile Zhang, Hongsheng Chen

**Affiliations:** 1 Interdisciplinary Center for Quantum Information, State Key Laboratory of Modern Optical Instrumentation, College of Information Science and Electronic Engineering, Zhejiang University, 310027 Hangzhou, China; 2 ZJU-Hangzhou Global Science and Technology Innovation Center, Key Lab. of Advanced Micro/Nano Electronic Devices & Smart Systems of Zhejiang, Zhejiang University, 310027 Hangzhou, China; 3 Department of Electrical Engineering, California Institute of Technology, Pasadena, CA USA; 4 ZJU-UIUC Institute, Zhejiang University, 310027 Hangzhou, China; 5 Division of Physics and Applied Physics, School of Physical and Mathematical Sciences, Nanyang Technological University, Singapore, 637371 Singapore

**Keywords:** Metamaterials, Electronics, photonics and device physics

## Abstract

Optical logic operations lie at the heart of optical computing, and they enable many applications such as ultrahigh-speed information processing. However, the reported optical logic gates rely heavily on the precise control of input light signals, including their phase difference, polarization, and intensity and the size of the incident beams. Due to the complexity and difficulty in these precise controls, the two output optical logic states may suffer from an inherent instability and a low contrast ratio of intensity. Moreover, the miniaturization of optical logic gates becomes difficult if the extra bulky apparatus for these controls is considered. As such, it is desirable to get rid of these complicated controls and to achieve full logic functionality in a compact photonic system. Such a goal remains challenging. Here, we introduce a simple yet universal design strategy, capable of using plane waves as the incident signal, to perform optical logic operations via a diffractive neural network. Physically, the incident plane wave is first spatially encoded by a specific logic operation at the input layer and further decoded through the hidden layers, namely, a compound Huygens’ metasurface. That is, the judiciously designed metasurface scatters the encoded light into one of two small designated areas at the output layer, which provides the information of output logic states. Importantly, after training of the diffractive neural network, *all seven* basic types of optical logic operations can be realized by the same metasurface. As a conceptual illustration, three logic operations (NOT, OR, and AND) are experimentally demonstrated at microwave frequencies.

## Metasurface provides clarity for optical logic

Carefully-designed diffractive metasurfaces enable accurate logical operations for optical computing. Using photons instead of electrons to convey information could reduce energy consumption and increase computing speed. However, the light properties must be controlled with very high precision in order to clearly distinguish between ‘0’ and ‘1’ logical states, and this is difficult to do without adding extra bulky components. Now, Hongsheng Chen at Zhejiang University in China and co-workers have used diffractive metasurfaces – carefully structured optical components designed from the atomic scale upwards – to realize all seven of the basic logic operations used in computation. Their components, which can be shrunk to the chip scale, clearly direct light into one of two designated areas indicating ‘0’ or ‘1’, even when the properties of the input light have not been optimally controlled.

## Introduction

Optical computing, which operates with photons instead of electrons, is becoming increasingly important, since it promises to increase the efficiency of information processing beyond traditional electron-based computing^1^. Due to its unique features of signal propagation at the speed of light, low power consumption, and the capability of parallel processing^2–5^, optical computing holds huge potential in many practical scenarios, particularly those involving high-throughput and on-the-fly data processing, such as augmented reality and autonomous driving^6^. The logic operation lies at the heart of all computers^7^. Correspondingly, optical logic gates^8–13^, including plasmonic logic gates, are essential for the further exploration and development of optical analogy computing, nanophotonic processing^14,15^, and the field of cryptographically secured wireless communication^16^. As such, there are growing and strong interests to provide optical logic gates with complete logic functionality in photonic systems with compact dimensions.

Previous methodologies towards optical logic gates considered mainly constructive/destructive interference effects, including linear^8–11^ and nonlinear interference^12,13^, between the input light signals. We note that the reported works are heavily dependent on the precise control of the basic properties of two input light signals, the control light and/or the pump light, including their phase difference, polarization, and intensity^7^ (Supplementary Note 6); if the two nanowires are close to each other, such as for the plasmonic logic gate, there is also a stringent requirement on the size of input light beams to avoid a potential false input. As a result, a better precise control of input light can more thoroughly realize constructive or destructive interference and lead to a larger intensity contrast ratio between the two output optical logic states “1” and “0”, which is a key feature to characterize the performance of an optical logic gate.

The heavy reliance on the precise control of input light has two unfavourable influences on the design of compact optical logic gates. First, their miniaturization becomes difficult if the additional bulky apparatus to achieve these controls are taken into consideration. Second, owing to the difficulty and complexity to achieve the ideal control of input light, their performance may suffer from an inherent instability, and the intensity contrast ratio between two output logic states may become quite low in practical scenarios^10^. For miniaturized optical logic gates, it is thus highly desirable to get rid of these critical requirements on the input light. Such a goal remains an open challenge that is long sought after due to its importance for the development of novel architectures for all-optical devices and systems.

To this end, here we introduce a simple yet universal design strategy, namely, a diffractive neural network^17^, to realize *all seven* basic optical logic operations in a compact system, simply using plane waves as the input signal. The diffractive neural network is implemented by a compound Huygens’ metasurface^18^, and it can partially mimic the functionality of an artificial neural network. After training, the compound metasurface can directionally scatter or focus the input encoded light into one of the two designated small areas/points, one of which represents logic state ‘1’ and the other stands for ‘0’. As a conceptual demonstration, three basic logic gates, i.e., NOT, OR, and AND, are experimentally verified using a two-layer high-efficiency dielectric metasurface at microwave frequency. Our design strategy features *two distinct advantages*. First, the realization of optical logic operations here gets rid of the complicated and necessarily precise control of the features of input light; such a scheme is thus totally different from previous works. Moreover, the design of the input layer is very general and powerful, and it can be flexibly modified into other user-favoured and programmable forms. Second, the proposed strategy can enable complete logic functionalities in a single optical network if the transmittance state of the input layer is dynamically tuneable, e.g., electrically tuneable if the optical mask is constructed by a spatial light modulator. Therefore, the revealed universal design strategy has the potential to facilitate a single miniaturized programmable photonic processor for arbitrary logic operations.

## Results

### Design principle and underlying physics of the optical logic operation

**Fig. 1:**
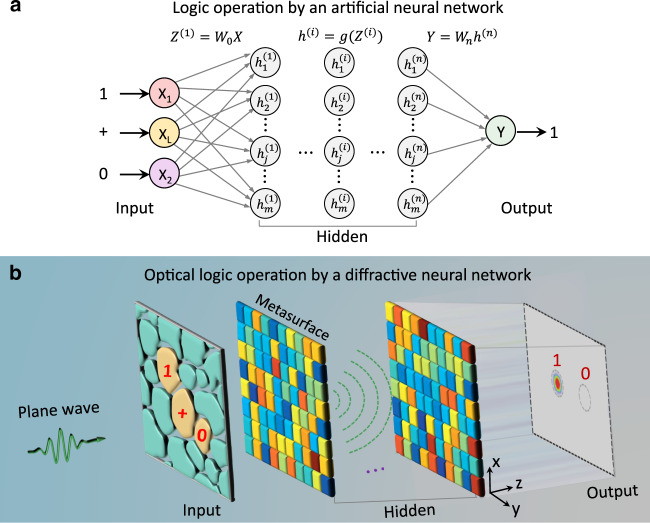
Schematic illustration of optical logic operations by a diffractive neural network. **a** Layout of a conventional artificial neural network for electron-based logic operations. **b** Layout of a diffractive neural network for photon-based logic operations. In **b**, each region at the input layer is assigned with a specific logic operator or an input logic state, and it has two different states for the transmittance of light. That is, the input layer can spatially encode the input plane wave for a specific optical logic operation, simply by setting the transmittance state of each region. The hidden layers, composed of metasurfaces, are designed to decode the encoded input light and generate an output optical logic state. In other words, the metasurface directionally scatters the encoded light into one of the two small designated regions at the output layer

We start with the design principle of the optical logic operation. For binary optical logic operation, the output has only two cases, ‘1’ or ‘0’, which is very similar to a classification/decision-making task from the perspective of machine learning^19^ and can be readily tackled by an artificial neural network; Supplementary Note 1 verifies the theoretical feasibility. Analogous to an artificial neural network (Fig. 1a), in the optical regime, a diffractive neural network (composed of one input layer, at least one hidden layer and one output layer) has been found to allow powerful wavefront manipulation and communicate information among layers at the speed of light. As delineated in Fig. 1b, the input layer is a common optical mask and is patterned to form multiple regions. Without loss of generality, each region in the optical mask is set to have two different states for the transmittance of light, and its high (low) transmittance state indicates that it is (is not) selected for optical computing. Then, it is possible and convenient to directly define all seven basic optical logic operators and the input logic states in the optical mask, simply by assigning each of them to a specific region. The hidden layers are designed to decode the encoded input light and image the calculated result at the output layer.

We then progress to the introduction of the underlying physics of the design of hidden layers. We use a metasurface made up of a dense array of subwavelength meta-atoms to construct each hidden layer. Each meta-atom behaves like an independent neuron in the neural network and interconnects to other meta-atoms of the following layers through the diffraction of light. Based on Rayleigh–Sommerfeld diffraction^20^, the meta-atom/neuron in the *l*th hidden layer, e.g., located at $\vec r_i^l = \left( {x_i^l,y_i^l,z_i^l} \right)$, serves as a secondary source. The Huygens wavelet of such a source arises as a *z*-derivative of the spherical wave (Fig. 1b) and can be described by $H_z^{{\mathrm {Huy}}}\left( {\vec r - \vec r_i^l} \right) = G\left( {\vec r_i^l} \right) \cdot h_z^{{\mathrm {Huy}}}\left( {\vec r - \vec r_i^l} \right)$, where

1 $$h_z^{{\mathrm {Huy}}}\left( {\vec r - \vec r_i^l} \right) = \frac{{ - 1}}{{2\pi }}\left( {ik - \frac{1}{R}} \right)\frac{{z - z_i^l}}{R}\frac{{{\mathrm {e}}^{ikR}}}{R}$$

In Eq. (1), $R = \sqrt {\left( {x - x_i^l} \right)^2 + \left( {y - y_i^l} \right)^2 + \left( {z - z_i^l} \right)^2}$, and *k* is the wavevector of light in free space. The complex-valued factor $G\left( {\vec r_i^l} \right)$ is determined by the product of the input wave $u\left( {\vec r_i^l} \right)$ to the neuron and its transmission coefficient $t(\vec r_i^l)$, i.e., $G\left( {\vec r_i^l} \right) = u\left( {\vec r_i^l} \right) \cdot t(\vec r_i^l)$. As such, the total propagation field $u\left( {\vec r} \right)$ is the summation of the field excited by all neurons in the *l*th layer, and it can be expressed as

2 $${\it{u}}\left( {\vec r} \right) = {{\int}\mathop {\int}\nolimits_{ - \infty }^\infty {H_z^{{\mathrm {Huy}}}\left( {\vec r - \vec r_i^l} \right){\it{{\mathrm {d}}x{\mathrm {d}}y}}} }$$

For the first hidden layer with *l* = 1, $u\left( {\vec r_i^1} \right)$ is the transmitted light spatially encoded by the input layer.

Following the forward propagation model in Eq. (2), the encoded input light can be directed into any desired location at the output layer via all learnable parameters $t(\vec r_i^l)$. As shown in Fig. 1b, we designate two small regions with a radius of less than half a wavelength. If most of the field intensity $s_i^{M + 1} = \left| {u\left( {\vec r_i^{M + 1}} \right)} \right|^2$ is focused in the left (right) region, the computing result is “1” (“0”). Note that this judgement criterion remains valid and consistent for all logic operations being considered, distinct from the case in refs. ^11,16^. Before implementing the diffractive neural network, the transmission coefficients $t\left( {\vec r_i^l} \right) = a_i^l \cdot {\mathrm {e}}^{i\phi _i^l}$ at each hidden layer should be adequately trained via an error back-propagation algorithm. In doing so, we define a loss function with mean square error $F\left( {t_i^l} \right) = \frac{1}{K}\mathop {\sum }\limits_i \left( {s_i^{M + 1} - g_i^{M + 1}} \right)^2$ to evaluate the performance between the output intensity $s_i^{M + 1}$ and the ground truth target $g_i^{M + 1}$, where *K* is the number of the measurement points. The gradient of the loss function with respect to all the trainable network variables is backpropagated to iteratively update the network during each cycle of the training phase until the network converges; see Supplemental Note 2 and “Methods” section for details. Note that, in our case, we do not split the input data into training, validation and test sets as done in the traditional manner, since our goal is to achieve zero-error classifications for all cases.

### Experimental demonstration of three basic logic operations, NOT, OR, and AND

**Fig. 2:**
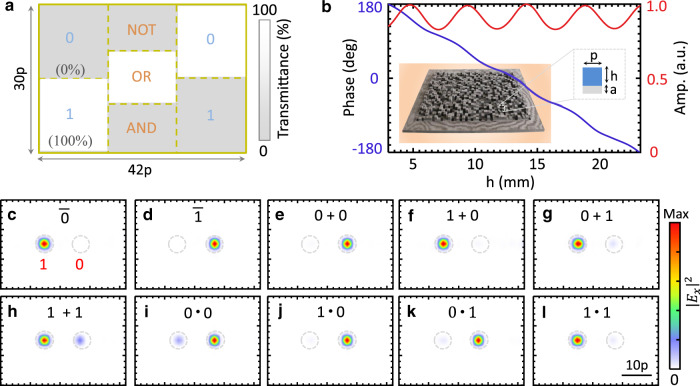
Numerical demonstration of three basic logic operations, i.e., NOT, OR, and AND, via a diffractive neural network. Here, the hidden layers are composed of two layers of metasurfaces. **a** Schematic of the input layer. The transmittance of light in the white (grey) region is set to be 100% (0%). The pattern in **a** indicates the optical logic operation of ‘1 + 0’; see more in Fig. S2. **b** Transmittance response of the metasurface, formed by a two-dimensional array of subwavelength meta-atoms. Each meta-atom can impart a local phase (blue line) and amplitude (red line) change to the input light. Each meta-atom has a square cross-section with a width of *p* = 10 mm and is deposited on a uniform dielectric slab with a thickness of *a* = 3 mm. **c**–**l** Intensity distribution at the output layer for three chosen logic operations with arbitrary input logic states. If the field is focused on the left (right) small designated regions, the output optical logic state is defined as “1” (“0”). The designated regions are highlighted by two dashed circles in each panel

As a conceptual demonstration, we first numerically realize three basic logic operations (Fig. 2), i.e., NOT, OR, and AND, at the designed frequency *f*_0_, since the combination of them can realize any other logic operation^9^. Our proposed design strategy for optical logic operations is, in principle, applicable for arbitrary frequencies. To facilitate the following experimental verification, *f*_0_ = 17 GHz (wavelength *λ*_0_ = 17.6 mm) is chosen here. Figure 2a shows the pattern of the input layer. For simplicity, the high (low) transmittance state for each region is assumed to have a transmittance of 100% (0%).

The hidden layers are composed of a cascaded two-layer transmission metasurface^21,22^ with an axial distance of 17*λ*_0_ (one of the tuneable parameters in the training process of diffractive neural network). Each metasurface consists of 30 × 42 meta-atoms (inset in Fig. 2b), where each meta-atom has a square cross section with a width of 0.57*λ*_0_. Here, we adopt a facile yet viable high-efficiency dielectric metasurface by taking advantage of its unique properties such as high transmittance and polarization insensitivity. The local transmission response of the designed meta-atoms is shown in Fig. 2b, where the constituent F4B dielectric has a relative permittivity of 3.5 + 0.003i and is fabricated by mechanical processing with an error <0.05 mm. The transmission phase *ϕ* varies smoothly over the height *h* of the meta-atom. Approximately, we have $h = \lambda _0\phi /2\pi \Delta n$, where Δ*n* is the refractive index difference between free space and the chosen dielectric. In contrast, the magnitude of transmission coefficients is almost uniform and close to unity. This way, one may target to train phase-only diffractive modulation layers. The training details are left to Supplementary Note 2. Figures 2c–l depict the numerical field intensity after training. As expected, most of the fields are correctly focused into one of the two small designated regions.

**Fig. 3:**
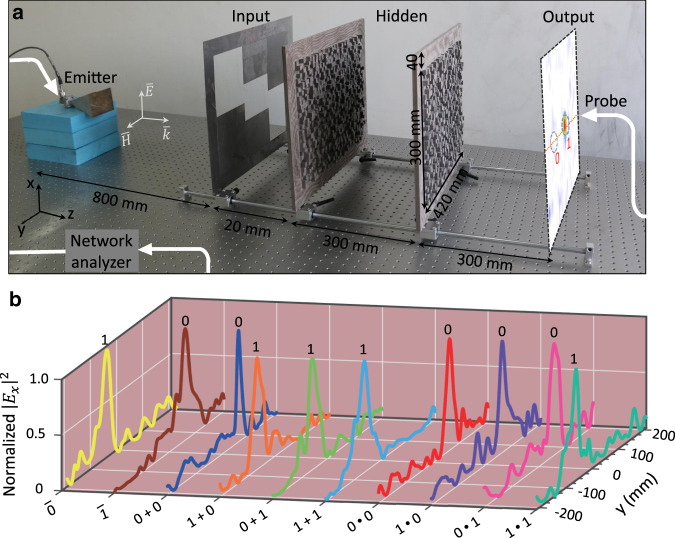
Experimental demonstration of three basic logic operations, i.e., NOT, OR, and AND, at a microwave frequency of 17 GHz. **a** Experimental setup. The metasurface has a cross section of 420 mm × 300 m, and to facilitate the installation, it is surrounded by a frame with a width of 40 mm. A small monopole probe moves automatically to scan the spatial intensity distribution at the output plane with a constant value of *z*. For example, the measured 2D scan for the logic operation of “1 + 0” is shown at the output plane in **a**. **b** Distribution of the measured normalized intensity along the dotted pink line with *x* = *x*_0_, namely, $\left| {E_x\left( {x_0,y} \right)} \right|^2/{\mathrm{max}}(\left| {E_x\left( {x_0,y} \right)} \right|^2)$, where *x*_0_ = 190 mm. All the maximum peaks are well confined within one of the two designated regions, consistent with the numerical simulations in Fig. 2

Figure 3 shows the microwave experimental demonstration of the theoretical proposal in Fig. 2. The experiment setup is depicted in Fig. 3a and described in “Methods” section. A horn antenna excites transverse electric (TE or *s*-polarized) waves with the electric field along the *x*-axis, and it is placed far from the input layer (~45*λ*_0_), so that the incident light signal can be reasonably treated as plane waves^23^ (see Fig. S5). The transmitted fields at the output layer, including their relative phase and amplitude, are measured by an E-field probe (a small monopole antenna^24^). For example, the inset at the output layer in Fig. 3a shows the measured 2D field intensity for the optical logic operation of “1+0”. Moreover, the experimental performance of all optical logic operations is shown in Fig. 3b. As expected, all the peaks of field intensity definitely appear within one of the two designated regions, consistent with Fig. 2c–l. Quantitatively, the contrast ratios between the measured intensities of two designated regions are all larger than 9.6 dB. The weak fields outside the two designated regions might be caused by the impedance mismatch at the air–dielectric interfaces, and this mismatch can be further reduced by introducing periodic antireflection structures^25^.

## Discussion

### Direct realization of all seven optical logic gates and cascaded optical logic gates

**Fig. 4:**
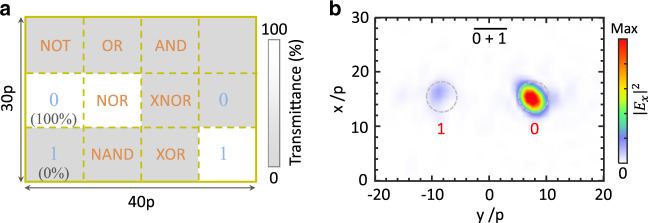
Numerical demonstration of all seven basic optical logic operations via a diffractive neural network. Here, the hidden layers are composed of three layers of metasurfaces. The design principle for the diffractive neural network follows Fig. 1 and is similar to Fig. 2. The space separation of successive layers is set to 22.7*λ*_0_ and *p* = 0.57*λ*_0_. **a** Schematic of the input layer. The pattern in **a** indicates the logic operation “$\overline {0 + 1}$”. **b** Intensity distribution for the logic operation of “$\overline {0 + 1}$” at the output layer. The intensity distribution for all other logic operations is shown in Fig. S6

We emphasize that the proposed design strategy can, in principle, directly construct any type (basic and compound) of optical logic operation, such as all seven basic logic operations as shown in Figs. 4 and S6. This can be done by extending the encoding manner at the input layer and developing a more sophisticated neural network configuration. For more complete functionalities, we can cascade multiple logic gates. As shown in Fig. S7, the output waves from one logic gate couple into the waveguides and then are guided to the input layer of another logic gate as the inputs^26^; see the details in Supplementary Note 5.

### Optical logic gates at higher frequencies

Although our experimental design in Fig. 3 only works at microwave frequencies, our theoretical design strategy in Fig. 1 should in principle be applicable to various frequency regimes, including terahertz and optical frequencies. The reason is that the main underlying mechanism in this work follows the universal diffractive law, which is scalable according to Maxwell equations. To let our proposed idea work at higher frequencies, we should at least consider scaling down the four key ingredients to higher frequencies, namely, the metasurfaces, the input light encoder (or the spatial light modulator), the light source and detector. These ingredients are accessible to experimental investigations with current technology^17,25,27^.

### Comparisons with the traditional-related design

Our design principles of a multi-functional optical logic gate and its switching behaviour are both different from those of the traditional related design; see Supplementary Note 6. First, the traditional multi-functional optical logic gate essentially relies on several single-functional logic gates, which are independent of each other and stacked for multi-functional capability. In contrast, our design relies on just one integrated multi-functional optical logic gate. Second, traditional switches generally need to precisely control the input light, or involve the nonlinearity and refractive indices of materials. These stringent controls unfavourably incur a high complexity, high cost, large volume, and even inherent instability of the system. In contrast, our switch gets rid of these stringent requirements, and it just allows or prevents light passing through the corresponding regions/channels. This simplified switch in our design makes a step towards a future miniaturized multi-functional optical logic gate.

### Other platforms to facilitate optical logic gates

Apart from the multi-layer metasurfaces, there are also other platforms to facilitate optical logic gates, for example, metamaterials/nanophotonics, which can offer ultra-high computing density in a compact and layer-free fashion^26^. By suitably engineering its spatial inhomogeneity, we can obtain an optical neural network on the chip scale, and some optical computing tasks such as image recognition and wavelength demultiplexer have already been facilitated^28^. In Fig. S9, we design a compact integrated-nanophotonic optical XOR logic gate as an example using topology optimization and finite-difference time domain (FDTD) simulation^29,30^.

To sum up, we have demonstrated a general framework for all optical logic operations by a compound Huygens’ metasurface enacted diffractive neural network, making a step towards multi-functional optical logic gates and high computing density. In a conceptually microwave experiment, we successfully realize three basic logical operations, i.e., NOT, OR, and AND, on a two-layer dielectric metasurface. Implementing our proposed architecture with metamaterials/nanophotonics may lead to chip-scale, ultrafast computing elements and promise the option of all-optical or hybrid optical–electronic technology. Looking forward, our proposed approach will also lead to a broad scope of applications, for example, real-time object recognition in surveillance systems and intelligent wave shaping inside biological tissues in microscope imaging^31^.

## Materials and methods

### Training of the diffractive neural network

The diffractive neural network is trained using Python version 3.5.0. and TensorFlow framework version 1.10.0 (Google Inc.) on a server (GeForce 249 10 GTX TITAN X GPU and Intel(R) Xeon(R) CPU X5570 @2.93 GHz with 48 GB RAM, running a Linux 250 operating system). It takes dozens of minutes to make our diffractive neural network converge. Notice that our process does not involve nonlinear activation function. We leave that to future work and experimentally compensate for its absence by a nonlinear optical medium, such as a photorefractive crystal and magneto-optical trap.

### Experiment setup

A near-field 3D scanning system was used for measurements. A horn antenna centred at the two-layer metasurface was used as the excitation source. Another small monopole probe oriented vertically to the ground was used to scan the relative amplitude and phase (S21) of the electric field *E*_*x*_. In measurement, the source and probe were connected to port 1 and port 2 of a vector network analyser, respectively, and the parameter S21 was recorded. The scan resolution in the *xoy* plane was 2 mm × 2 mm.

## Supplementary information

Supplementary Material
