# Supplementary material for: Performing optical logic operations by a diffractive neural network
Source: Light Sci Appl. 2020 Apr 13;9:59. doi: 10.1038/s41377-020-0303-2 (PMC7154031; doi:10.1038/s41377-020-0303-2)
Supplement: Supplementary file 1 — Supplementary Material [file 41377_2020_303_MOESM1_ESM.docx]

Supplementary Information for

**Performing optical logic operations by a diffractive neural network**

Chao Qian^1,2,3,4^, Xiao Lin^5,*^, Xiaobin Lin^1^, Jian Xu^3^, Yang Sun^1,2^, Erping Li^1,4^,

Baile Zhang^5,*^, and Hongsheng Chen^1,2,4*^

*^1^ Interdisciplinary Center for Quantum Information, State Key Laboratory of Modern Optical Instrumentation, College of Information Science and Electronic Engineering, Zhejiang University, Hangzhou 310027, China.*

*^2^ ZJU-Hangzhou Global Science and Technology Innovation Center, Key Lab. of Advanced Micro/Nano Electronic Devices & Smart Systems of Zhejiang, Zhejiang University, Hangzhou 310027, China.*

*^3^ Department of Electrical Engineering, California Institute of Technology, Pasadena, CA, USA.*

*^4^ ZJU-UIUC Institute, Zhejiang University, Hangzhou 310027, China.*

*^5^ Division of Physics and Applied Physics, School of Physical and Mathematical Sciences, Nanyang Technological University, Singapore 637371, Singapore.*

*^*^Corresponding author:* [*xiaolinbnwj@ntu.edu.sg*](mailto:xiaolinbnwj@ntu.edu.sg) *(X. Lin);* [*blzhang@ntu.edu.sg*](mailto:blzhang@ntu.edu.sg) *(B. Zhang);* [*hansomchen@zju.edu.cn*](mailto:hansomchen@zju.edu.cn) *(H. Chen)*

**The PDF file includes:**

- Supplementary Note 1: Verification that logic operations can be addressed by neural network
- Supplementary Note 2: Gradient descent of the diffractive neural network
- Supplementary Note 3: Experimental calibration
- Supplementary Note 4: Direct realization of all seven optical logic gates
- Supplementary Note 5: Cascaded optical logic gates
- Supplementary Note 6: Comparisons with the traditional related design
- Supplementary Note 7: Other platforms to facilitate optical logic gates

**Supplementary Note 1: Verification that logic operations can be addressed by neural network**

In essence, all logical operations can be understood as a classification task with three dimensionalities $(X_{1},X_{2},X_{L})$, where $X_{1},X_{2}$ indicate the input binary number, and $X_{L}$ indicates the logic type such as NOT, OR, and AND. In the following, we would like to verify that such classification task can be addressed by artificial neural network from the perspective of *theory*.

For conceptual clarity, we start from a two-dimensional $(X_{1},X_{2})$ case, as shown in Fig. S1a, and divide the whole space into two classes, $C_{1}$ and $C_{2}$; assuming $C_{1}$ contains a triangle and a curved region. If an input $(X_{1},X_{2})$ is located in $C_{1}$, the output $Y=1$, otherwise $Y=0$, equivalent to the case of logical operation. By the backward propagation algorithm, we can obtain all boundary functions, such as the three edges of the triangle, i.e., $l_{1}$, $l_{2}$ and $l_{3}$, which is crucial to distinguish $C_{1}$ and $C_{2}$. Thus, an input point $P$ located in the triangle should simultaneously satisfy three conditions ①$z_{1}^{(1)}\geq0$，②$z_{2}^{(1)}\geq0$, ③$z_{3}^{(1)}\geq0$, and after a process by nonlinear activation function $g$, $h_{1}^{(1)}=h_{2}^{(1)}=h_{3}^{(1)}=1$. By setting the weight $w_{1}^{(2)}=w_{2}^{(2)}=w_{3}^{(2)}=1$ and bias $b_{1}^{(2)}=-2.5$, it ensures that, only when the three conditions are all met, $h_{1}^{(2)}=1$. For the curved region, we can epitomize it by a sufficiently large number of boundary functions and treat the corresponding weights $w_{4}^{(2)}=w_{5}^{(2)}=\ldots w_{j}^{\left( 2 \right)}\ldots=w_{m}^{(2)}=1$ and bias $b_{2}^{(2)}=-(m-3.5)$ in the same way.

Furthermore, by simply setting $w_{1}^{(3)}=w_{2}^{(3)}=1$ and $b_{1}^{(2)}=-0.5$, we find that, the final output $Y=1$ for an input point either in the triangle or the curved region. In other words, only when the input point is outside of both the triangle and the curved region, the output $Y=0$. In light of the above weight setting and theoretical analysis, we can conclude that such classification task can be addressed by neural network. Very similarly, it is readily generalized into three-dimensional $(X_{1},X_{2},X_{L})$ classification task of the logical operation in our work.

**
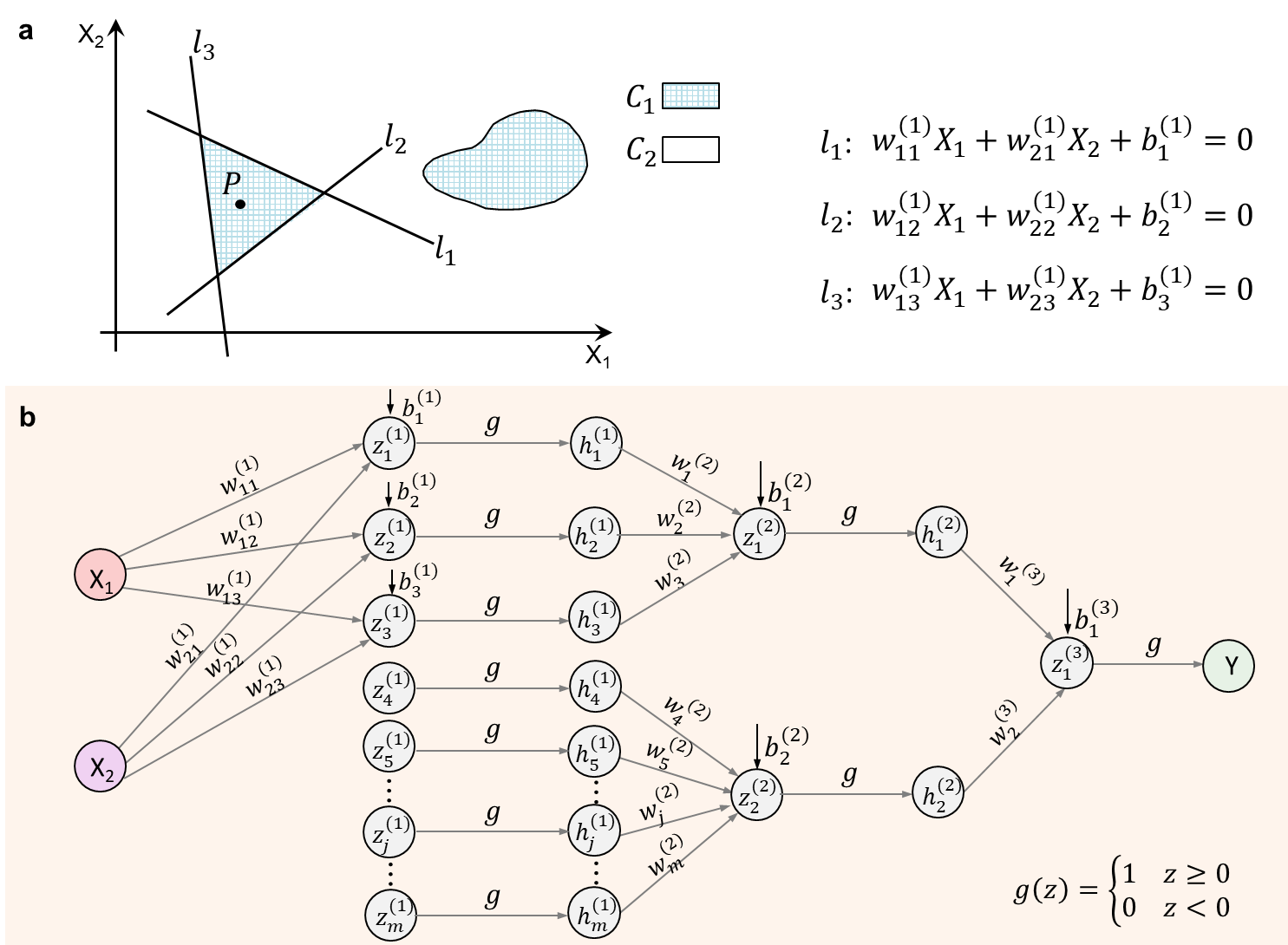
**

**Fig. S1 | Verification that logic operations can be addressed by neural network. a**, Two-dimensional space with two categories $C_{1}$ and $C_{2}$. **b**, Neural network to address the classification task of the logic operation.

**Supplementary Note 2: Gradient descent of the diffractive neural network**

Our optimization objective is to minimize the loss function and can be written as:

$\min F\left( t_{i}^{l} \right), s.t. 0\leq\phi_{i}^{l}\leq2\pi, 0\leq a_{i}^{l}\leq1$ (S1)

where $t_{i}^{l}=a_{i}^{l}\cdot e^{i\phi_{i}^{l}}$. The gradient of $F\left( t_{i}^{l} \right)$ with respect to $t_{i}^{l}$ at a given layer $l$ is expressed as:

$\frac{\partial F\left( t_{i}^{l} \right)}{\partial t_{i}^{l}}=\frac{4}{K}\sum_{k} \left( s_{k}^{M+1}-g_{k}^{M+1} \right)\cdot real\{\left( u_{k}^{M+1} \right)^{*}\cdot\frac{\partial u_{k}^{M+1}}{\partial t_{i}^{l}}\}$ (S2)

Based on Eq. (2) in the main text, $u_{k}^{M+1}$ can be discretized as $u_{k}^{M+1}=\sum_{k1} h_{k1}^{M}\left( x_{k},y_{k},z_{k} \right)\cdot t_{k1}^{M}\left( x_{k1},y_{k1},z_{k1} \right){\cdot u}_{k1}^{M}\left( x_{k1},y_{k1},z_{k1} \right)$. Thus, for $l=M$,

$\frac{\partial u_{k}^{M+1}\left( x_{k},y_{k},z_{k} \right)}{\partial t_{i}^{l=M}}=\frac{\partial\sum_{k1} h_{k1}^{M}\left( x_{k},y_{k},z_{k} \right)\cdot t_{k1}^{M}\left( x_{k1},y_{k1},z_{k1} \right){\cdot u}_{k1}^{M}\left( x_{k1},y_{k1},z_{k1} \right)}{\partial t_{i}^{l=M}}$ (S3)

Simplifying this expression,

$\frac{\partial u_{k}^{M+1}\left( x_{k},y_{k},z_{k} \right)}{\partial t_{i}^{l=M}}=t_{i}^{M}\left( x_{i},y_{i},z_{i} \right){\cdot u}_{i}^{M}\left( x_{i},y_{i},z_{i} \right)\cdot h_{i}^{M}\left( x_{k},y_{k},z_{k} \right)$ (S4)

To be specific, when we consider a spatial phase-only metasurface, i.e., the transmission amplitude is uniform,

$\frac{\partial u_{k}^{M+1}\left( x_{k},y_{k},z_{k} \right)}{\partial\phi_{i}^{l=M}}=i\cdot t_{i}^{M}\left( x_{i},y_{i},z_{i} \right){\cdot u}_{i}^{M}\left( x_{i},y_{i},z_{i} \right)\cdot h_{i}^{M}\left( x_{k},y_{k},z_{k} \right)$ (S5)

Similarly, for $l=M$-1,

$\frac{\partial u_{k}^{M+1}\left( x_{k},y_{k},z_{k} \right)}{\partial t_{i}^{l=M-1}}=\frac{\partial\sum_{k1} h_{k1}^{M}\left( x_{k},y_{k},z_{k} \right)\cdot t_{k1}^{M}\left( x_{k1},y_{k1},z_{k1} \right)\cdot\sum_{k2} n_{k2}^{M-1}\left( x_{k1},y_{k1},z_{k1} \right)}{\partial t_{i}^{l=M-1}}$ (S6)

$$\frac{\partial u_{k}^{M+1}\left( x_{k},y_{k},z_{k} \right)}{\partial t_{i}^{l=M-1}}=t_{i}^{M-1}\left( x_{i},y_{i},z_{i} \right)\cdot u_{i}^{M-1}\left( x_{i},y_{i},z_{i} \right)\cdot$$

$\sum_{k1} h_{k1}^{M}\left( x_{k},y_{k},z_{k} \right)\cdot t_{k1}^{M}\left( x_{k1},y_{k1},z_{k1} \right)\cdot w_{i}^{M-1}\left( x_{k1},y_{k1},z_{k1} \right)$ (S7)

Following this rule, we can obtain the partial derivatives for other hidden layers $1\leq l\leq M$-2 using chain derivation. In a general form, we can write,

$\frac{\partial u_{k}^{M+1}\left( x_{k},y_{k},z_{k} \right)}{\partial t_{i}^{l=M-A}}=t_{i}^{M-A}\cdot u_{i}^{M-A}\cdot\sum_{k_{1}} h_{k1,k}^{M}\cdot t_{k1}^{M}\cdots\cdots\sum_{k_{A}} h_{k_{A},k_{A}-1}^{M-A+1}\cdot t_{k_{A}}^{M-A+1}\cdot h_{i,k_{A}}^{M-A}$ (S8)

where $2\leq A\leq M$-1. During each iteration of the error backpropagation, the training data is fed into the optical neural network to generate the loss function, then used to update the whole neural network until convergence, i.e., the loss does not decline. To reduce the potential coupling effect of the metasurface in the experiment, a sigmoid function is used to squash the phase coverage by half, i.e., the transmission phase $\phi_{i}^{l}$ varies within $0-\pi.$

In the design of the three basic logical operations, the input plane is segmented into three columns and seven subregions; Fig. S2 shows some examples of the input pattern. Figure S3 shows the training loss over epochs and the final training phase-only masks. Afterwards, we construct the phase mask using a high-efficiency dielectric metasurface and numerically simulate it by CST Microwave Studio. As shown in Fig. S4, the incident wave is molded into the expected region, in agreement with Fig. 2f of the main text.


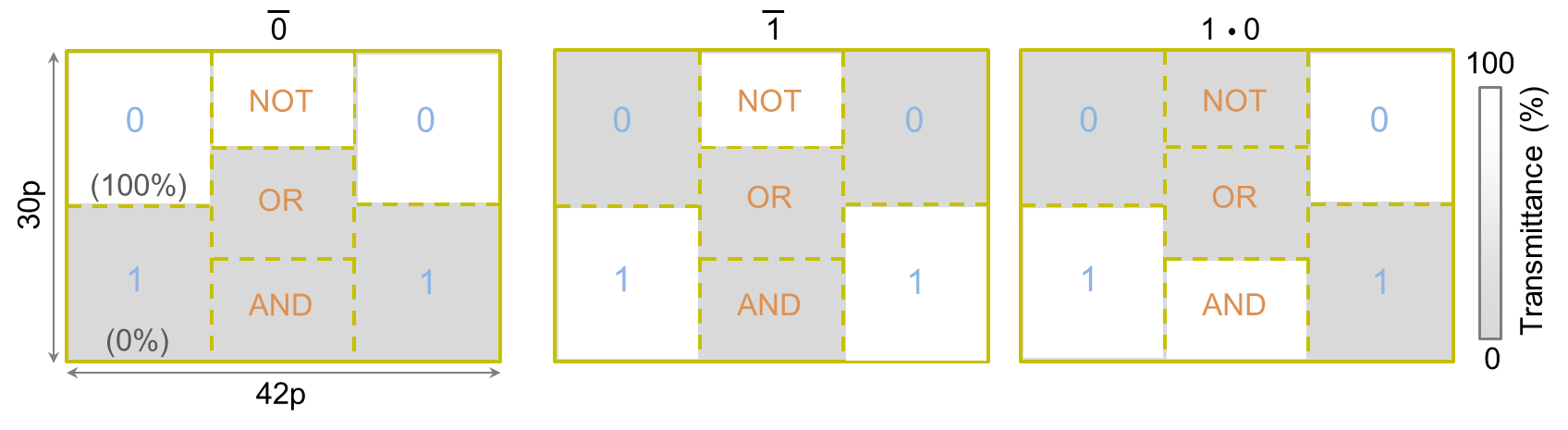


**Fig. S2 | Input light encoding of** $\bar{\boldsymbol{0}}$**,** $\bar{\boldsymbol{1}}$ **and** $\boldsymbol{1\cdot0}$**.**


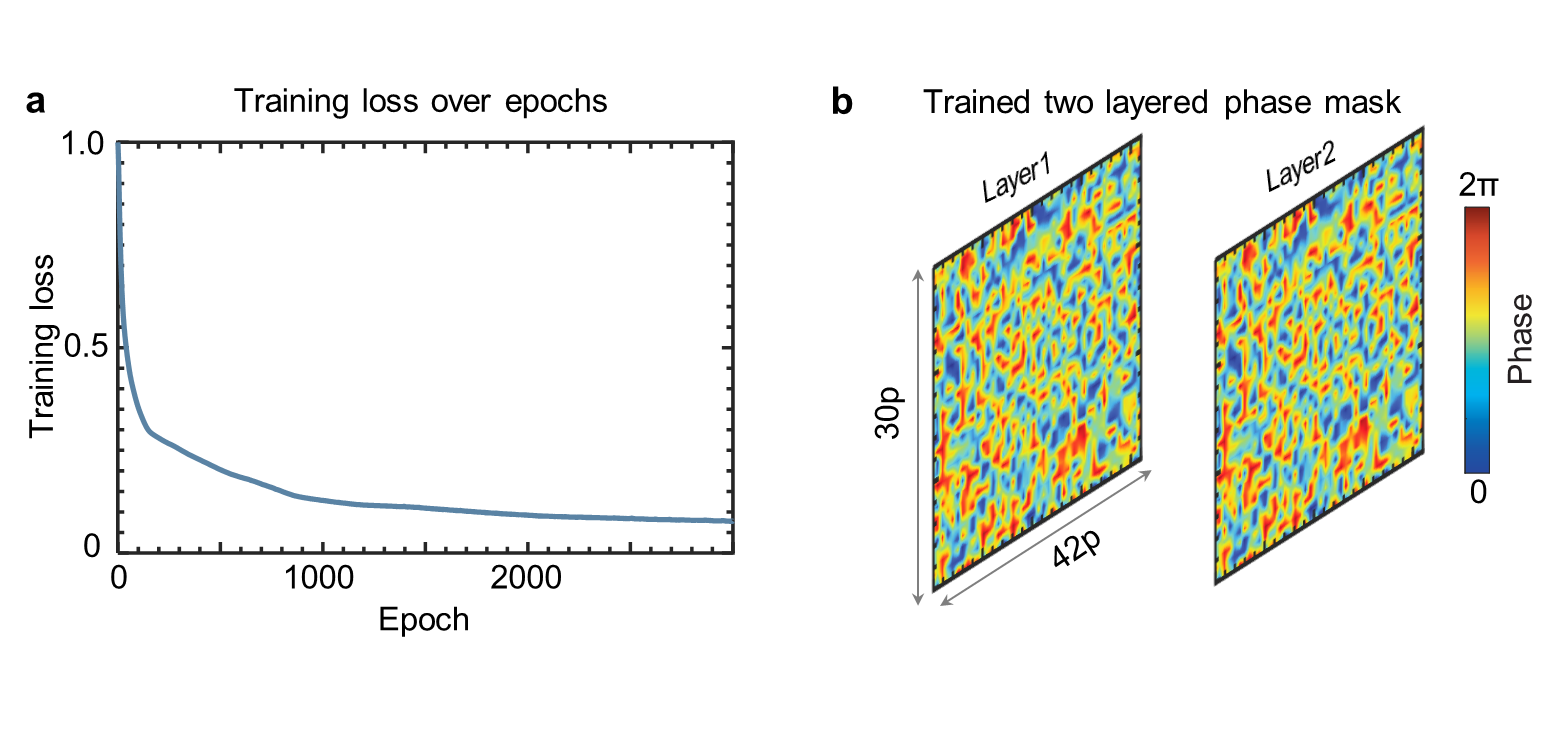


**Fig. S3 | Training loss and the final spatial phase distributions.**

**
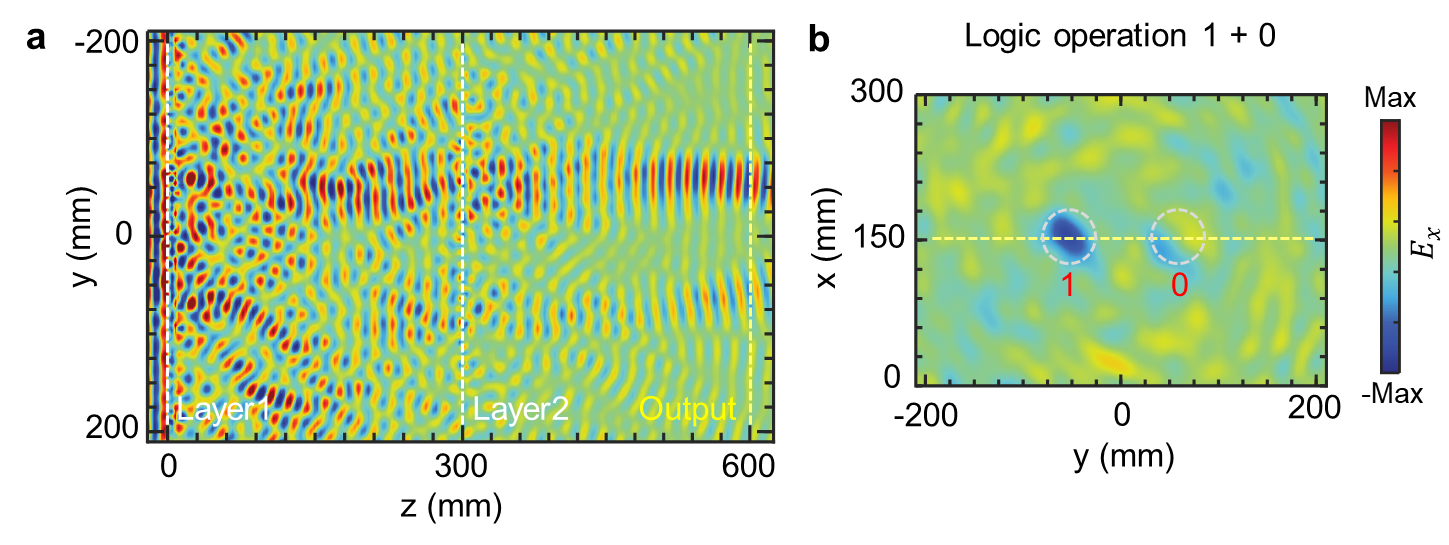
**

**Fig. S4 | Numerical simulation of the logic operation ‘1+0’.**

**Supplementary Note 3: Experimental calibration**

As marked in Fig. 3a, the horn antenna is located 800 mm (approximately ${45\lambda}_{0}$, where $\lambda_{0}$ is the free-space wavelength at the working frequency 17 GHz) away from the first layer or plane. In such a far-field region, the incident wave can be reasonably treated as plane waves [23]. The experimental results in Fig. S5 corroborate such a claim, which shows the uniform distribution of the measured incident waves at the first layer.


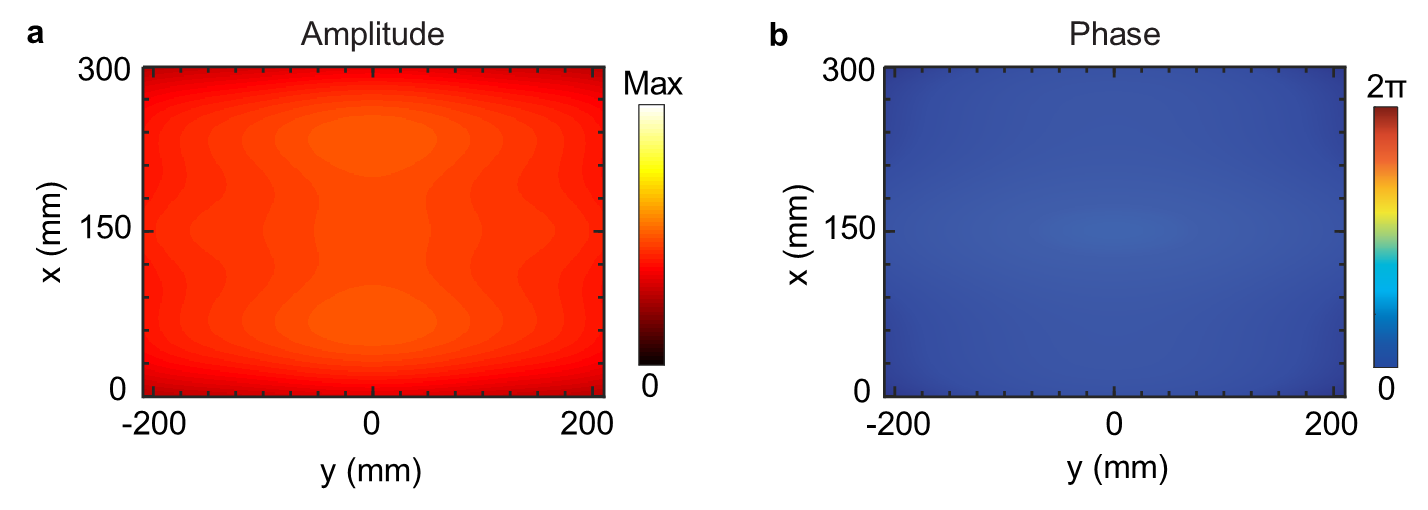


**Fig. S5 | Distribution of the measured amplitude (a) and phase (b) of incident waves at the first layer.** Their uniform distribution indicates that the excited waves transmit equally to the first layer in Fig. 3a.

**Supplementary Note 4: Direct realization of all seven optical logic gates**

Similar to Fig. 2 in the main text, here we utilize a three-layer phase-only diffractive neural network to realize all seven optical logic gates in a single optical system. As shown in Fig. 4 of the main text, the whole input plane ($30p_{0}\times40p_{0}$) is divided into twelve uniform subregions, where $p_{0}=0.57\lambda_{0}$. The axial distance between two successive layers is set to $22.7\lambda_{0}$. For all logic operations, we calculate the intensity distributions of the two designated regions in Fig. S6, with satisfied accuracy.


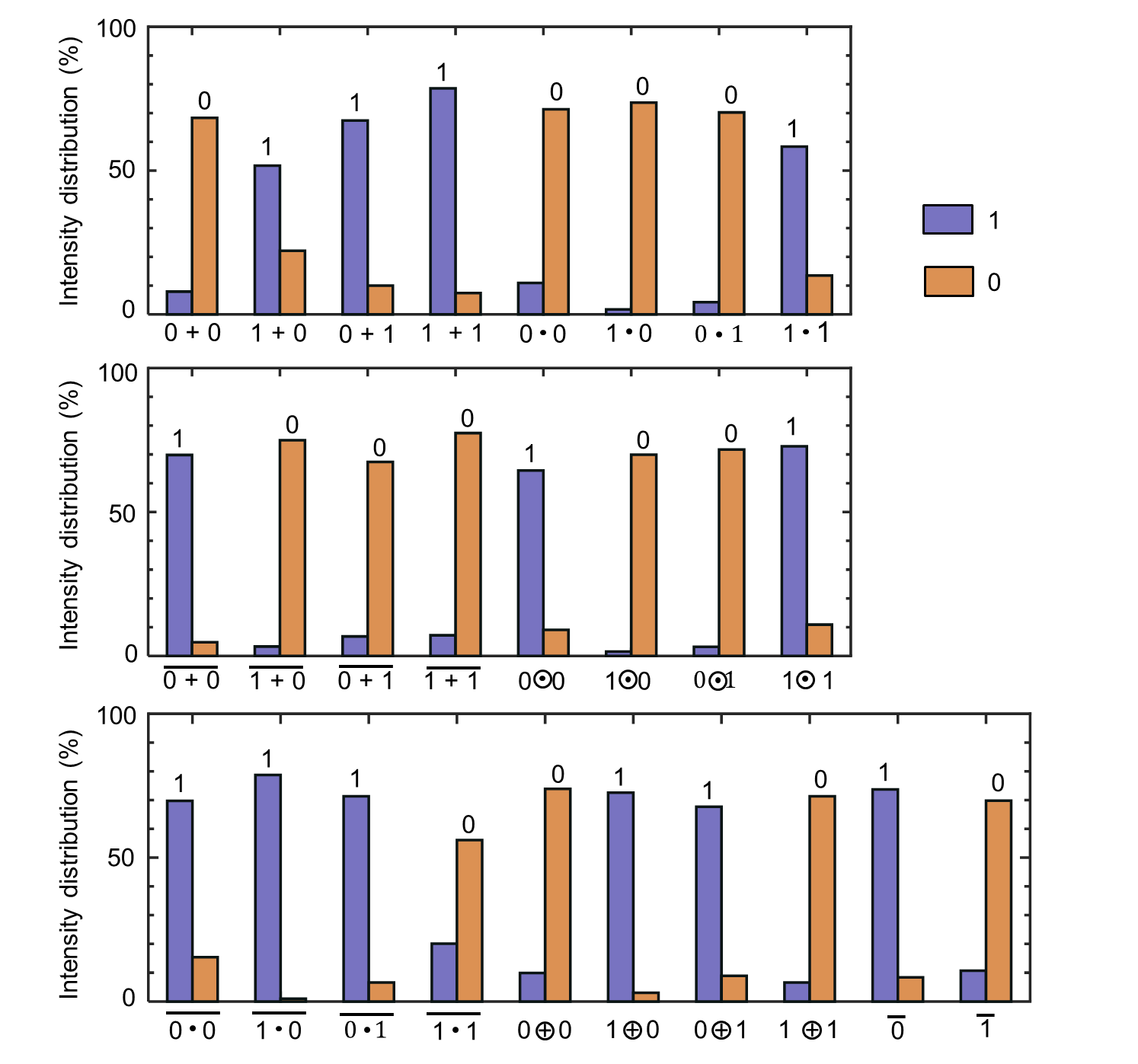


**Fig. S6 | Numerical results of all seven optical logical operations.**

**Supplementary Note 5: Cascaded optical logic gates**

The possible scheme to realize the cascading of optical logic gates is illustrated in Fig. S7, by following the recent publication [26]. To be specific, the output waves from logic gates 1 and 2 firstly couple into the waveguides and then are guided to the input layer of logic gate 3 as the inputs. In practice, the coupling efficiency of waves between the waveguide and the output or input layer maybe not unitary. Such an uncertainty or imperfection will not degrade the performance of logic gates, and it can be readily calibrated or tackled during the design of logic gates. As a conceptual demonstration, Fig. S7b-d shows the numerical performance of two-level cascaded logic gate. The numerical results corroborate the feasibility of our proposed scheme in Fig. S7a.

Similarly, we can construct optical register to realize bit saving and copying, like the electronic counterparts. For example, the optical RS flip-flop (a one-bit memory device) can be set up by a pair of cross-coupled two-unit NAND gates. The NAND gate has been conceptually demonstrated in Fig. 4 of the main text.


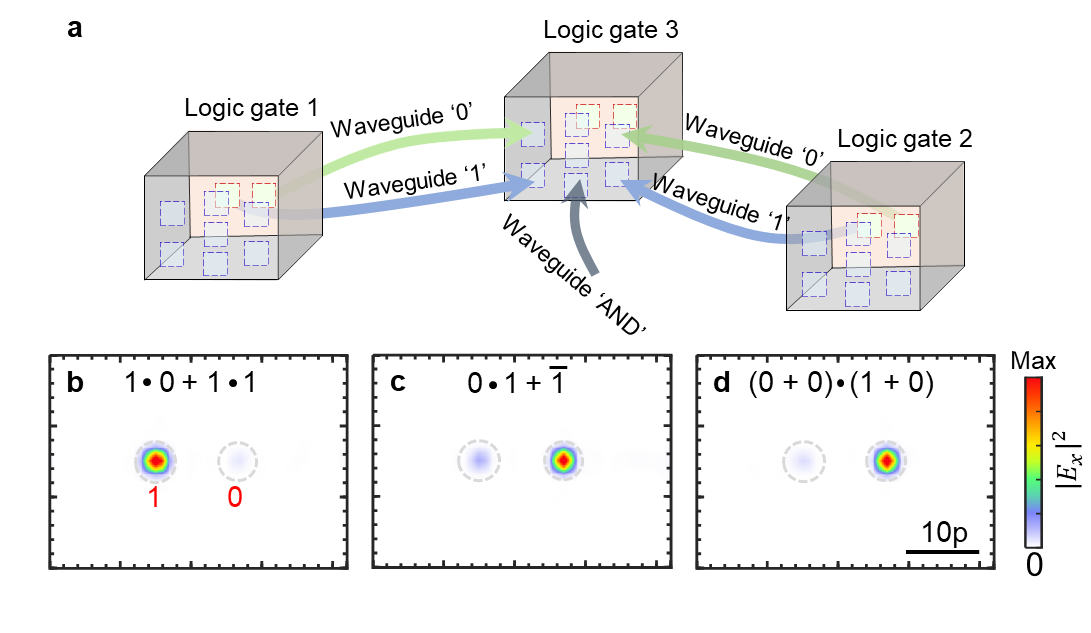


**Fig. S7 | Cascaded logic gates.** **a**, Schematic illustration of cascaded logic gates. Each logic gate is symbolized by a small box. The outputs of logic gates 1 and 2 are interconnected to the inputs of the logic gate 3 via waveguides. Waveguide ‘AND’ is to activate the AND function of logic gate 3. The other two functions, namely NOT and OR, can be activated in a similar way. **b**-**d**, Intensity distributions at the output layer of the logic gates 3 for three logic operations. For conceptual demonstration, we assume that the output waves of logic gates 1 and 2 can be safely (e.g., 100%) guided by the waveguide to the prescribed regions at the input layer of the logic gate 3. Herein, all the parameters of logic gates are consistent with those in the main text. p is the period of the unit cell for the metasurface.

**Supplementary Note 6: Comparisons with the traditional related design**

Our design principle of a multi-functional logic gate and its “switch” manner are both different from those of the traditional design, as schematically illustrated in Fig. S8.

First, we discuss about the design principle of multi-functional logic gate. The traditional design in Fig. S8a essentially relies on *three* single-functional logic gates, which are independent of each other and are stacked together for multi-function capability. In contrast, our design in Fig. S8b relies on just *one* integrated multi-functional logic gate. We highlight that the three logic gates (i.e., NOT, AND and OR) in Fig. S8b share the same and fixed metasurfaces. Second, we proceed to the discussion of the “switch” manner for multi-functional logic gates. For the traditional design, the switch, for example, can be pumping light. It generally requires precise control of the phase, polarization and intensity of input light, or the nonlinearity and refractive indices of materials. These stringent controls unfavorably incur a high complexity and high cost in the design, and moreover, it may lead to a large volume for the whole system and even some inherent system instability. In contrast, the switch in our design gets rid of these stringent requirements. To be specific, the switch in our design is simply realized by allowing or preventing the light passing through the corresponding regions/channels. Taking “1+0” as an example, the switch in our design just needs to allow the light to pass through “0”, “+”, “1” channels at the input layer. This simplified switch in our design makes a step towards a future miniaturized multi-functional optical logic gate.


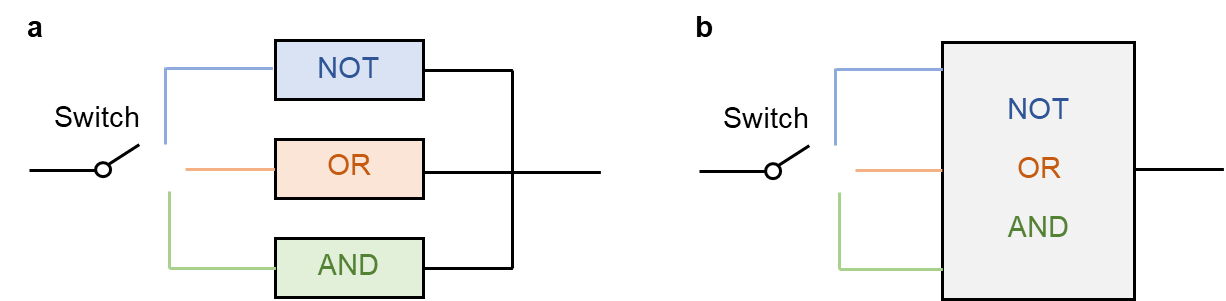


**Fig. S8 | Comparisons with traditional related design.** **a**, Traditional design of multi-functional optical logic gate, namely “a switch plus three independent single-functional logic gates”. **b**, Integrated multi-functional optical logic gates in this work. The three logic gates in (**b**) share the same and fixed metasurfaces. Moreover, the switch manner for the logic gates in (**a**) and (**b**) are totally different.

**Supplementary Note 7: Other platforms to facilitate optical logic gates**

In addition to the multilayered metasurfaces, there are also other platforms to facilitate optical logic gates, for example, metamaterials/nanophotonics. As shown in Fig. S9a, we design a compact integrated-nanophotonic optical XOR logic gate, which consists of two input waveguides, one square computational region (inverse designed using topology optimization) [S1,S2], and one output waveguide. Illustrated in (Figs. S9(b-d)) are the simulated energy distributions and all optical XOR logic operations are correct. More speculatively, we can envision a potential possibility that the output of the optical logic gate can be directly cascaded to the input of other optical logic gate by optical waveguide or forming a feedback optical network for many exciting applications [26, S3].


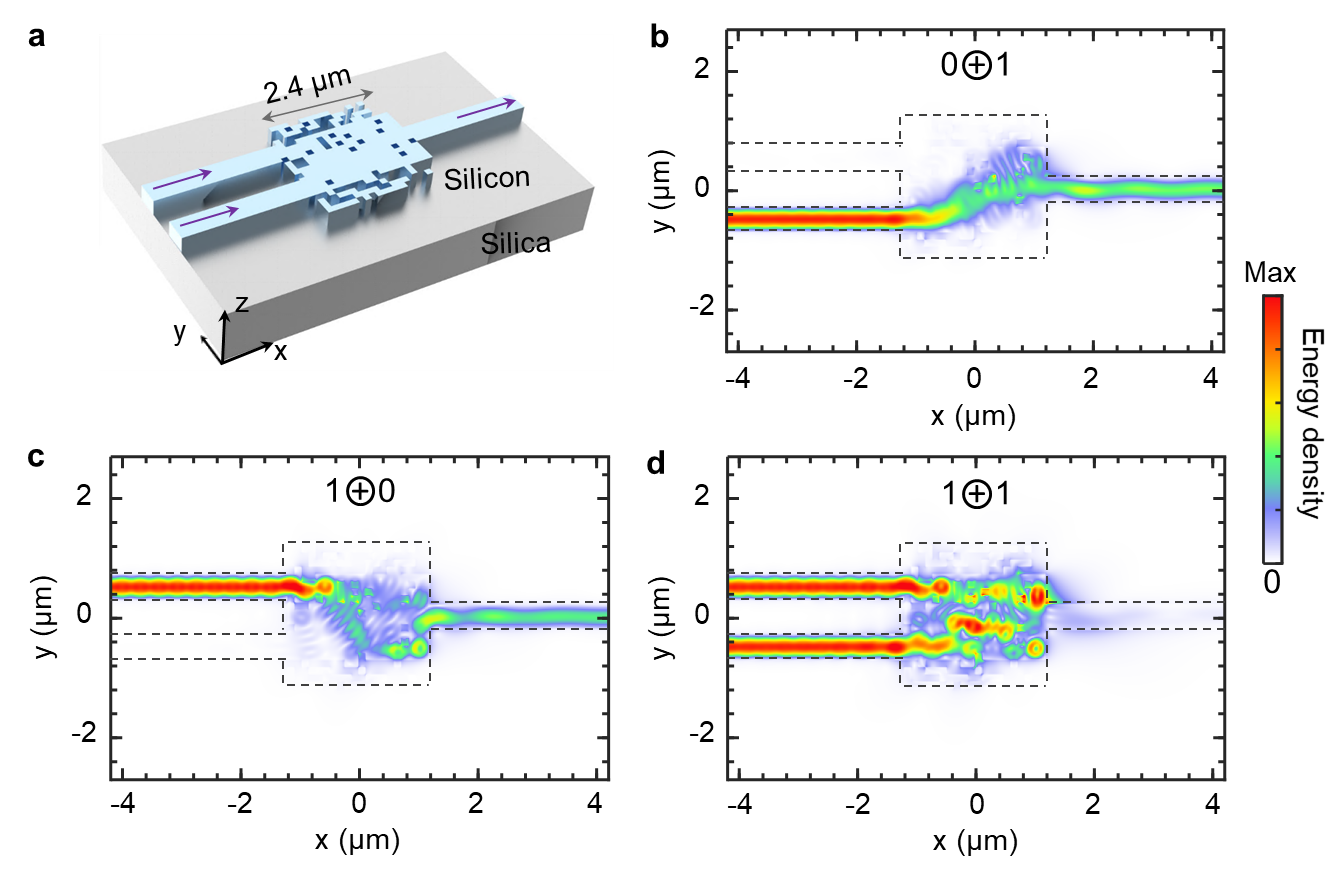


**Fig. S9 | On-chip nanophotonic optical logic operation. a**, Three-dimensional illustration of the optical logic gate consisting of two parallel input waveguides separated by 1.0 μm, one computational region with 2.4×2.4 μm^2^ footprint, and one output waveguide. All the three silicon waveguides are identical with the width of 440 nm and thickness of 300 nm. Here the fundamental TE wave (which is polarized in-plane and perpendicular to the propagation direction) is considered as both the input and output modes, for the inverse design of the central computational region by topology optimization method. As an example, the optical logic operation of XOR is designed and simulated by finite-difference time domain (FDTD) solver [29,30]. **b**-**d**, Energy density distributions at 1,550 nm for 0 XOR 1, 1 XOR 0, and 1 XOR 1, respectively, and the three calculated results are all correct. Note that the operation result of 0 XOR 0 is obviously zero and thus it is not presented.

**References**

[S1] Shen, B. *et al.* An integrated-nanophotonics polarization beamsplitter with 2.4 × 2.4 μm^2^ footprint. *Nat. Photon.* **9**, 378-382 (2015).

[S2] [Piggott](https://www.nature.com/articles/nphoton.2015.69#auth-1), A. Y. *et al.* Inverse design and demonstration of a compact and broadband on-chip wavelength demultiplexer. *Nat. Photon.* **9**, 374-377 (2015).

[S3] Khoram, E. *et al.* Nanophotonic media for artificial neural inference. *Photon. Res.* **7**, 823-827 (2019).
